# *Mycobacterium africanum* (Lineage 6) shows slower sputum smear conversion on tuberculosis treatment than *Mycobacterium tuberculosis* (Lineage 4) in Bamako, Mali

**DOI:** 10.1371/journal.pone.0208603

**Published:** 2018-12-12

**Authors:** Bassirou Diarra, Mahamadou Kone, Antieme Combo Georges Togo, Yeya dit Sadio Sarro, Aissata Boubakar Cisse, Amadou Somboro, Boureima Degoga, Mohamed Tolofoudie, Bourahima Kone, Moumine Sanogo, Bocar Baya, Ousmane Kodio, Mamoudou Maiga, Michael Belson, Susan Orsega, Meryam Krit, Sounkalo Dao, Ibrahim Izétiegouma Maiga, Robert L. Murphy, Leen Rigouts, Seydou Doumbia, Souleymane Diallo, Bouke Catherine de Jong

**Affiliations:** 1 University Clinical Research Center (UCRC)-SEREFO-Laboratory, University of Sciences, Techniques and Technologies of Bamako (USTTB), Bamako, Mali; 2 Institute of Tropical Medicine, Department of Biomedical Sciences, Antwerp, Belgium; 3 Department of Biomedical Sciences, Antwerp University, Antwerp, Belgium; 4 Laboratoire National de Référence des Mycobactéries (LNR), Institut National de Recherche en Santé publique (INRSP), Bamako, Mali; 5 Global Health, Northwestern University, Chicago, IL, United States of America; 6 Collaborative Clinical Research Branch, Division of Clinical Research, National Institute of Allergy and Infectious Diseases, Bethesda, Maryland, United States of America; 7 Laboratoire d’analyses Médicales et Hygiène Hospitalière du Centre Hospitalier Universitaire du Point-G, Bamako, Mali; St Petersburg Pasteur Institute, RUSSIAN FEDERATION

## Abstract

**Objective:**

Ancestral *M*. *tuberculosis* complex lineages such as *M*. *africanum* are underrepresented among retreatment patients and those with drug resistance. To test the hypothesis that they respond faster to TB treatment, we determined the rate of smear conversion of new pulmonary tuberculosis patients in Bamako, Mali by the main MTBc lineages.

**Methods:**

Between 2015 and 2017, we conducted a prospective cohort study of new smear positive pulmonary tuberculosis patients in Bamako. Confirmed MTBc isolates underwent genotyping by spoligotyping for lineage classification. Patients were followed at 1 month (M), 2M and 5M to measure smear conversion in auramine (AR) and Fluorescein DiAcetate (FDA) vital stain microscopy.

**Result:**

All the first six human MTBc lineages were represented in the population, plus *M*. *bovis* in 0.8% of the patients. The most widely represented lineage was the modern Euro-American lineage (L) 4, 57%, predominantly the T family, followed by L6 (*M*. *africanum* type 2) in 22.9%. Ancestral lineages 1, 5, 6 and *M*. *bovis* combined amounted to 28.8%. Excluding 25 patients with rifampicin resistance, smear conversion, both by AR and FDA, occurred later in L6 compared to L4 (HR 0.80 (95% CI 0.66–0.97) for AR, and HR 0.81 (95%CI 0.68–0.97) for FDA). In addition we found that HIV negative status, higher BMI at day 0, and patients with smear grade at baseline ≤ 1+ were associated with earlier smear conversion.

**Conclusion:**

The six major human lineages of the MTBc all circulate in Bamako. Counter to our hypothesis, we found that patients diseased with modern *M*. *tuberculosis* complex L4 respond faster to TB treatment than those with *M*. *africanum* L6.

## Introduction

With an estimated 1.7 million deaths worldwide, tuberculosis (TB) remains one of the leading causes of death from an infectious disease[1]. Its causative pathogen, the *M*. *tuberculosis* complex (MTBc), consists of seven geographically diverse lineages of human importance in addition to the animal lineages that occasionally spill over into humans[2]. Each of the lineages is associated with a specific global geographical distribution[2]. Human lineage (L) 6, also named *M*. *africanum* West African 2, is the closest relative of the animal lineages of the MTBc. Lineage 6 is endemic in West Africa, where it causes up to a third of pulmonary TB[3,4]. Lineage 5 (*M*. *africanum* West African 1) is endemic in a region more to the east (South-Central West Africa), with overlap in countries like Ghana and Benin[3]. Lineage 6 was found to progress to disease slower, and to be overrepresented in HIV-infected hosts[3,5,6] in some studies, but not others[7,8]. Among West African migrants in the U.S., L5 and L6 are overrepresented in extrapulmonary disease[5]. Together, L1, L5 and L6 are considered ‘ancestral lineages’, as opposed to the ‘modern’ MTBc lineages L2, L3, and L4.

Most studies on the MTBc population structure in the West African region were based on convenience sampling at referral centers, rather than sampling representative of the general TB population[9,10]. This potential for selection bias, combined with the higher risk for L6 to be missed in culture, has complicated estimates of the prevalence of L6 over time[10,11]. In several countries, L6 appears to be declining, while in others its prevalence appears to be stable, with most recent estimates for Mali suggesting a prevalence of between 21 and 33.3% in 1994, 27.8% in 2012, and 16.8% between 2008 and 2016 in Mali[4,10,12].

The treatment for L6 is the same as for other human lineages, with typically good programmatic outcomes on Category 1 treatment (two months (M) of rifampicin (R), isoniazid (H), pyrazinamide (Z) and ethambutol (E) and 4M of R and H (2RHZE/4RH). Good programmatic outcome is defined as smear conversion at 5M by fluorescent Auramine Rhodamine (AR) or Ziehl Neelsen (ZN) microscopy, although a fair proportion of patients who still have AR smear positive sputum at 5M have negative cultures, i.e. they are excreting dead bacilli[13], which can be differentiated from live bacilli by fluorescein diacetate (FDA) vital stain microscopy. In such patients, the persistently positive AR smear is likely reflective of a higher bacterial burden at baseline rather than bacteriological failure.

*M*. *africanum* (L6) is underrepresented among retreatment patients and those with drug resistance[3,4,10,14]. However, some studies have found that the immunological recovery of patients treated for L6 lags behind those treated for L4[15,16]. Given this uncertainty on the response of L6 to treatment originally developed for ‘modern’ MTBc lineages[17,18], we set out to test- in a population based prospective study- the hypothesis that patients with AFB smear positive pulmonary TB caused by L6, convert their smears faster than those diseased with L4, in classical AR as well as FDA vital stain microscopy.

## Materials & methods

### Study design and setting

Between 2015 and 2017, we conducted a prospective cohort study, enrolling consecutive new adult smear positive pulmonary tuberculosis patients (PTB) from five local TB diagnostic and treatment centers in Bamako, Mali. This was an observational study enrolling tuberculosis patients after providing written informed consent.

Bamako, the capital city, has a population of approximately two million people, about 14% of the population of Mali, living in six urban districts, with each district having a health referral center, where TB diagnostic and treatment services are available. In 2015 alone, more than one third of the total TB patients in Mali (7,015 patients) were managed in Bamako, and our study population represents more than 60% of the eligible patients in participating health centers.

The study protocol was approved by the Ethics Committee of the University of Sciences, Techniques, and Technologies of Bamako (FMOS/FAPH), the institutional review board (IRB) of the Institute of Tropical Medicine and the University of Antwerp, Belgium, and the IRB of WHO/TDR, Geneva, Switzerland.

### Study population

**Fig 1:**
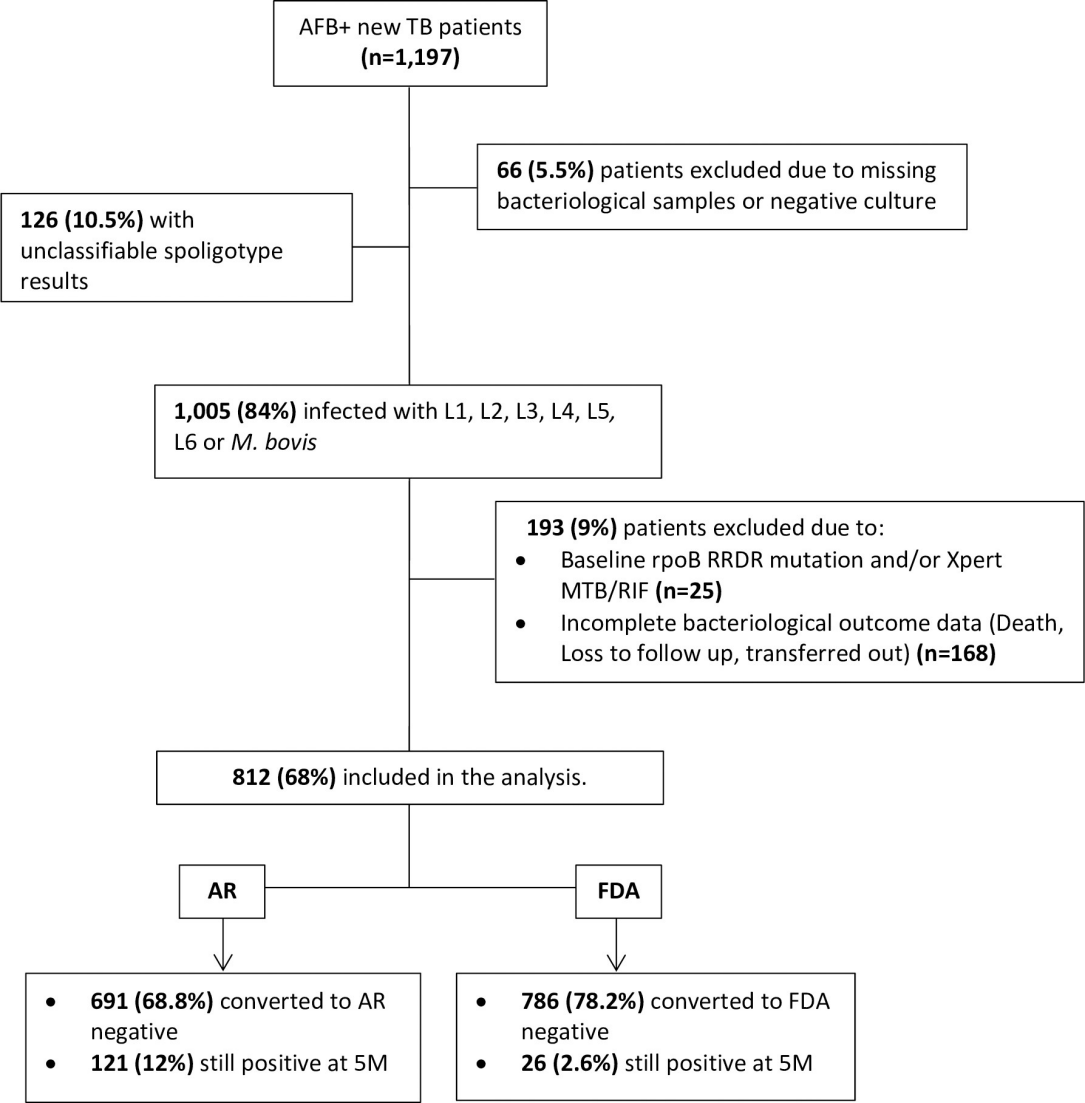
Flow chart of tuberculosis patients enrolled and included in final analysis. L = lineage 1, 2, 3, 4, 5 and 6; F = *M*. *tuberculosis* family 33, 34, and 36; AR = Fluorescent auramine/rhodamine staining technique; FDA = Fluorescein di-acetate vital staining technique.

Consecutive patients with presumptive pulmonary TB had sputum screened at the study sites by either Ziehl Neelsen (ZN) or Auramine/Rhodamine (AR) staining. Adult (age ≥ 18) smear positive consenting participants were enrolled into the study (Fig 1). Patients were treated in accordance with national guidelines of the TB program in Mali[19], in which new patients receive a fixed dose combination of 2RHZE/4RH. After baseline screening (D0) just before starting TB treatment, and at 1M, 2M and 5M, fresh sputum samples were collected for microscopy. Body mass index (BMI) was calculated by dividing the weight in kilogram (Kg) at each visit by the square of the height in centimeters (cm^2^) measured at baseline.

### Laboratory tests

Pre-enrollment sputum smear microscopy by ZN or direct FM using AR (BBL Becton Dickinson, Sparks MD, USA) at local reference centers was followed by FDA staining and culture at the UCRC BSL-3 laboratory, which is certified by the college of American pathologists (CAP), including external quality controls.

### Culture and identification

Sputum specimens were digested and decontaminated using the standard N-Acetyl-L-Cysteine/4% NaOH solution, concentrated by centrifugation (3000g) and inoculated on both liquid (*Mycobacterium* Growth Incubator Tube (BBL MGIT Becton Dickinson, Sparks MD, USA)), and solid (Middlebrook 7H11 Agar and Selective 7H11 Agar) media. Simultaneously, an aliquot of concentrated sediment was prepared for indirect microscopy by AR and FDA. Speciation of positive mycobacterial cultures was based on AFB-positivity in microscopy and colony morphology on solid medium, and was confirmed by Capilia TB Test (TAUNS Laboratories, Numazu, Japan), or by nucleic acid probes (AccuProbe GenProbe, San Diego, CA, USA).

### Indirect microscopy staining protocols

In addition to ZN, we used FDA vital staining, a much less widely used stain, in spite of recognition of its utility since 1992. In FDA staining living cells stain fluorescent green due to their ability to enzymatically hydrolyze (through active esterases) non fluorescent fluorescein diacetate to fluorescent fluorescein. The presence of an intact cell membrane permits the bacteria to intracellularly accumulate fluorescein[20].

*The grading of sputum smear microscopy* was done following the International Union Against Tuberculosis and Lung Diseases (IUATLD) grading criteria as negative (No AFB seen or 0), scanty AFB seen (1–9 AFB per 100 immersion fields), few AFB seen (10–99 AFB seen in 100 fields or 1+), Moderate AFB seen (1–10 AFB seen per field or 2+), and Many AFB seen (>10 AFB seen per field or 3+)[21]. To minimize inaccuracies, two technicians evaluated each smear and in case of discrepancy a third person was asked to resolve.

### Molecular strain typing

Baseline isolates had *rpoB* sequenced to determine rifampicin resistance (RR) a posteriori, while some of these patients were identified as having RR-TB in Xpert at 2M or 5M. Spoligotyping was performed on boiled bacterial lysates using a commercially available kit (Ocimum)[22] or by in-house prepared membrane. Data entry from the film was verified by a second assessor. Lineage classification was based on the SPOTCLUST (SpolDB3-based, http://tbinsight.cs.rpi.edu/run_spotclust.html) database, TB Miner http://info-demo.lirmm.fr/tbminer/index.php; and the L6 specific polymorphism in *rpoB* at codon 388 described in Bamako previously [23].

### HIV testing

The human immunodeficiency virus (HIV) serology status was determined using previously described methods[24–26].

### Data & statistical analysis

Only new TB patients who were sputum smear positive and had *Mycobacterium tuberculosis* complex (MTBc) disease confirmed by culture were included in the final analysis. Those with known resistance to the most powerful TB drug, rifampicin, were excluded.

We fitted Weibull proportional-hazards regression to measure the effects of predictors such as BMI, type of MTBc lineage, smear grade, and HIV on the hazard rate (HR). Univariate and multivariate analyses were conducted with demographic and clinical variables to identify predictors of smear conversion relative to different lineages. Results were considered significant when p-values were less than 0.05.

Survival analysis was based on the smear conversion by AR or FDA staining as outcome, by L4 versus L6.

## Results

Of the 1,197 patients enrolled through December 2017, 1,131 (94.5%) had an isolate available for typing (Fig 1). The prevalence of HIV coinfection was 9.4%. Most patients (78.6%) were between 18 and 44 years old, and 72.5% of all patients were male. More than half of patients (52.2%) were under weight (Baseline BMI < 18.5 kg/m^2^).

### Lineage and family assignments

All the first six lineages of human importance were represented in the *M*. *tuberculosis* complex population structure in Bamako, in addition to 0.8% *M*. *bovis*. The most widely represented lineage was the modern L4, (57%), predominantly the T family, followed by L6 (*M*. *africanum* type 2, 22.9%). Furthermore, L1 (Indo-oceanic, 3.7%), L2 (East Asia including Beijing, 1.4%), L3 (Central Asia, 1.6%), and L5 (*M*. *africanum* type 1.4%) were also identified (Table 1). Patterns identified as Family 33, 34, or 36 could not be assigned to a phylogenetic lineage and constituted 11.1%.

**Table 1:** Distribution of major *M*. *tuberculosis* complex families (Lineages 1–6) and *M*. *bovis* from a total of 1,131 strains from newly infected TB patients in Bamako, Mali.

Lineage (L)	Family	n (%) of total isolates
Indo-oceanic (L1)	EAI	42(3.7%)
East Asian (L2)	Beijing	16(1.4%)
Central Asia (L3)	CAS	18(1.6%)
Euro-American (L4)	LAM	242(21.4%)
	Haarlem	81(7.2%)
	T-Clade	276(24.4%)
	H37Rv-like	3(0.3%)
	S-Clade	17(1.5%)
	X-Clade	26(2.3%)
West African 1 (L5)	MAF-1	16(1.4%)
West African 2 (L6)	MAF-2	259(22.9%)
Animal lineage (*M*. *bovis*)		9 (0.8%)
Unclassifiable (families 33, 34, 35, 36)		126 (11.1%)

Global distribution of the major six lineages identified in Bamako between February 2015, and December 2017.

Patient characteristics did not differ significantly between those infected with L4 versus L6. HIV coinfection was non-significantly higher among ancestral lineages, including *M*. *bovis* at 22.2%, L1 at 14.7%, and L5/L6 at 10.8%, compared to 7.5% of L4 (HIV prevalence in combined *M*. *bovis*, L1, L5, and L6 relative to modern lineage (L4) (OR 1.52 (95% CI 0.94–2.44).

### Smear conversion

Of the 1,005 patients with interpretable Spoligotyping results, 812 (80.8%) were included in the final smear conversion analysis (Fig 1). Patients presented with high bacterial loads (73.8% had ≥3+ AFB in AR smear and 44.6% had ≥3+ AFB in FDA smear), which differed by lineage: baseline AR smears were ≥3+ in 77% of L6 patients relative to 72.6% of L4, while FDA smears were ≥3+ in 51% of L6 patients versus 42.3% for L4. Only nine patients (0.95%) were FDA negative at baseline.

Controlled for baseline smear grade, HIV, and BMI, AFB-smear conversion to negative by AR occurred faster for L4 than for the other lineages, with L6 the slowest (HR of 0.80 relative to L4 (95% CI 0.66–0.97)) (Table 2). Also, conversion of vital staining by FDA was delayed in lineage 6 compared to lineage 4 (HR 0.81 (95%CI 0.68–0.97)) (Table 3).

**Table 2:** Weibull proportional hazard regression to measure the effects of predictors such as BMI, type of lineages, and HIV on the hazard rate using auramine conversion time.

	*Unadjusted*			*Adjusted*		
Lineages	Haz. Ratio	*p*	*[95% CI]*	Haz. Ratio	*p*	*[95% CI]*
1	1.09	0.63	0.76–1.56	1.09	0.67	0.72–1.65
2	1.04	0.89	0.58–1.84	1.00	0.98	0.53–1.89
3	0.70	0.27	0.37–1.31	0.58	0.19	0.26–1.31
5	0.80	0.48	0.42–1.49	1.15	0.68	0.57–2.32
6	0.79	0.01	0.66–0.95	0.80	0.02	0.66–0.97
*M*. *bovis*	0.53	0.15	0.22–1.27	0.59	0.25	0.24–1.44
HIV positive	0.48	0.00	0.35–0.66	0.45	0.00	0.32–0.63
BMI (day 0)	1.05	0.00	1.03–1.08	1.06	0.00	1.03–1.09
Few AFB (day 0)	0.45	0.03	0.22–0.95	0.44	0.03	0.21–0.93
Moderate and Many AFB (day 0)	0.35	0.00	0.17–0.72	0.34	0.00	0.17–0.70

Proportions of smears converted and non-converted by Lineage to estimate the effect of predictors in smear conversion using Auramine Rhodamine staining technique; L = lineage 1, 2, 3, 5 and 6; HIV = Human Immunodeficiency Virus; BMI = Body Mass Index; CI = Confidence Interval; p = Probability. L4 is not shown as it is the reference for the others. In addition HIV+ was fitted against HIV-, and AFB smear grade were fitted against smear negative.

**Table 3:** Weibull proportional hazard regression to measure the effects of predictors such as BMI, type of lineages, and HIV on the hazard rate using fluorescein di-acetate conversion time.

	*Unadjusted*			*Adjusted*		
Lineages	Haz. Ratio	*p*	*[95% CI]*	Haz. Ratio	*p*	*[95% CI]*
1	0.95	0.79	0.67–1.34	0.96	0.85	0.64–1.43
2	1.25	0.40	0.73–2.13	1.21	0.51	0.66–2.22
3	0.65	0.14	0.36–1.15	0.57	0.12	0.28–1.16
5	0.96	0.89	0.55–1.67	1.75	0.06	0.95–3.19
6	0.80	0.00	0.67–0.94	0.81	0.02	0.68–0.97
*M*. *bovis*	0.49	0.09	0.22–1.11	0.53	0.12	0.23–1.18
HIV positive	0.50	0.00	0.37–0.66	0.47	0.00	0.34–0.64
BMI (day 0)	1.04	0.00	1.02–1.07	1.05	0.00	1.02–1.08
Few AFB (day 0)	0.48	0.05	0.23–1.00	0.44	0.03	0.21–0.93
Moderate and Many AFB (day 0)	0.42	0.01	0.20–0.84	0.40	0.01	0.20–0.81

Proportions of smear converted and non-converted per Lineages to estimate the effect of predictors in smear conversion using Fluorescein di-acetate vital staining technique; L = lineage 1, 2, 3, 4, 5 and 6; HIV = Human Immunodeficiency Virus; BMI = Body Mass Index; CI = Confidence Interval; p = Probability. L4 is not shown as it is the reference for the others. In addition HIV+ was fitted against HIV-, and AFB smear grade were fitted against smear negative.

At 5M, of the 121 patients with positive AR, 71(58.7%) were diseased with L4, and 38 (31.4%) with L6, while of 691 negative AR patients at 5M, 456 (66%) were L4 and 165 (23.9%) L6 (OR 0.68 (95% CI 0.43–1.04)).

Similarly, of the 26 patients with positive FDA at 5M, 13(56.5%) were L4, and 7(26.9%) were L6, whereas of 786 negative FDA, 511(65%) were diseased with L4, and 195 (24.8%) with L6 (OR 0.72 (95% CI 0.27–1.80))

In addition, we found that HIV negative status, and higher baseline BMI, were associated with faster smear conversion using both techniques (Tables 2 and 3). Furthermore, and as expected, patients who had many (3+) or moderate (2+) AFB seen at day 0, had slower conversion compared to patients with few AFB (1+) and to patients with no AFB seen using both techniques (Tables 2 and 3).

**Fig 2:**
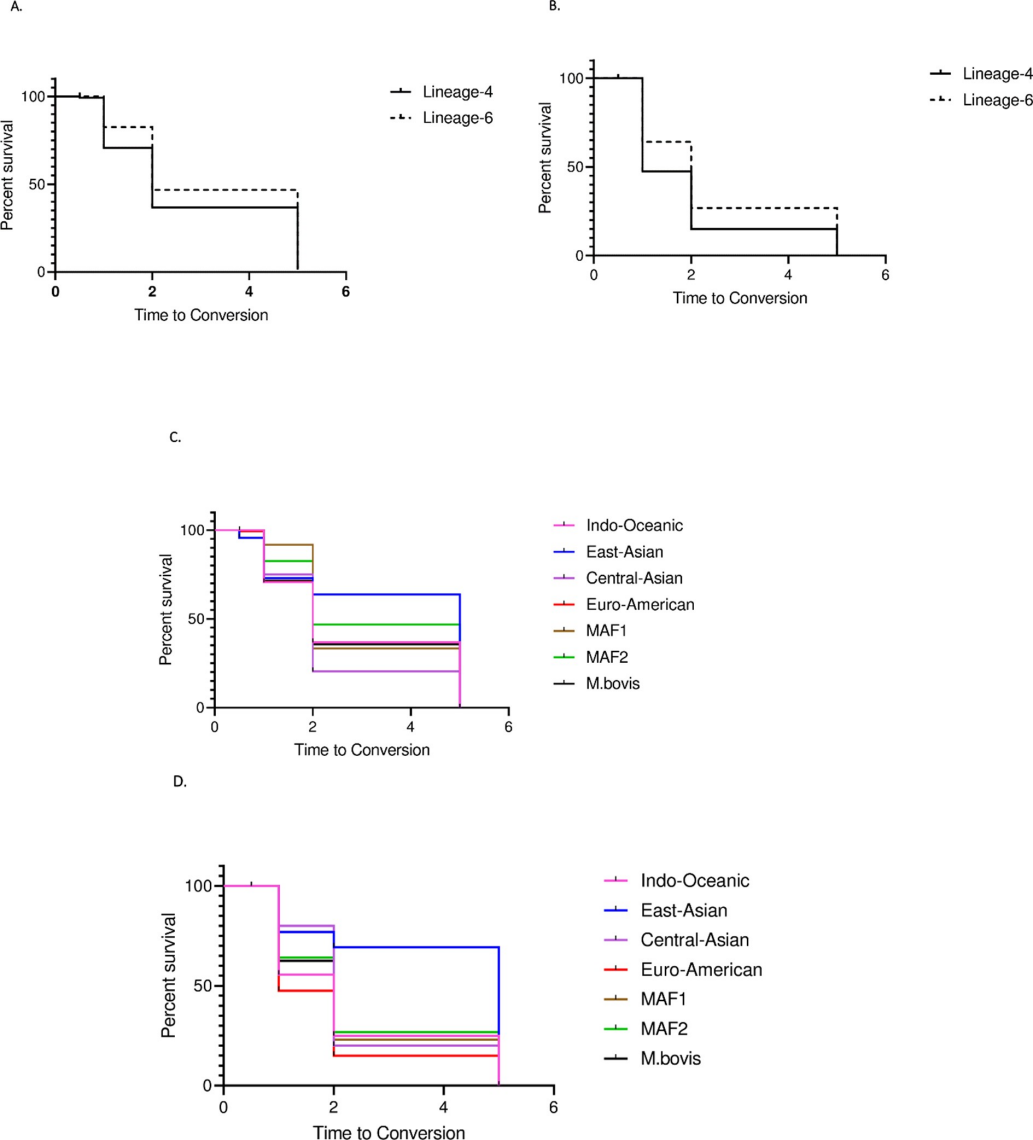
**A. Time to conversion of L4 versus L6 as measured by Auramine Rhodamine.** Survival analysis of smear conversion by AR staining as outcome between Lineage 4 (Euro-American) and Lineage 6 (MAF2), p = 0.0066. MAF2 = *Mycobacterium africanum* type 2 L4 = 645; L6 = 259 **B. Time to conversion of L4 versus L6 as measured by Fluorescein DiAcetate** Survival analysis of smear conversion by FDA staining as outcome between Lineage 4 (Euro-American) and Lineage 6 (MAF2), p<0.0001. MAF2 = *Mycobacterium africanum* type 2; L4 = 645; L6 = 259 **C. Time to conversion by lineage as measured by Auramine Rhodamine** Survival analysis of smear conversion by AR staining as outcome between Lineage (L) 1, L2, L3, L4, L5 and L6, p = 0.06. L1 = 42 patients; L2 = 16; L3 = 18; L4 = 645; L5 = 16; L6 = 259; *M*. *bovis* = 9 **D. Time to conversion by lineage as measured by Fluorescein DiAcetate** Survival analysis of smear conversion by FDA staining as outcome between Lineage (L) 1, L2, L3, L4, L5 and L6, p<0.01 L1 = 42 patients; L2 = 16; L3 = 18; L4 = 645; L5 = 16; L6 = 259; *M*. *bovis* = 9.

Survival analysis confirmed that in both techniques sputum smear conversion of patients diseased with L6 lagged behind those of Lineage 4 at 1-month period (1M), 2M and 5M (Fig 2A and 2B).

## Discussion

In this study we estimated a relatively unbiased population structure of the MTBc in consecutive smear positive new adult TB patients from Bamako district, and identified differential smear conversion by MTBc lineage. Our prevalence estimates of the different MTBc lineages correspond with earlier and smaller studies based on convenience sampling, with predominance of L4 (57%) and L6 (22.9%), while L1, L2, L3, and L5 are all present at less than five percent[4,10]. Counter to our hypothesis that L6 would respond better to TB treatment, we found that patients diseased with L6 rather experienced slower smear conversion than L4, not only with AR staining- which will stain dead bacilli too- but also with FDA vital stain microscopy.

Tuberculosis patients in Bamako clearly present with advanced disease, with three quarters having 3+ AFB on baseline AR smear examination. As expected, we showed that patients who presented with ≥ 2+ AFB had slower conversion compared to those with ≤ 1+ AFB.

Given that the standard TB treatment in use today was largely developed in India and Europe, rather than in L6 endemic regions, this could explain why patients with L4 respond earlier to standard treatment than those with L6. If L6 converts slower, it may also remain transmissible longer, which may explain why L6 is still prevalent in the West African region despite its underrepresentation among patients with resistant TB [3,4,6]. In spite of their later conversion, L6 patients were not significantly more likely to be AR or FDA positive at 5M than L4 patients (OR 1.54 (95% CI 0.89–5.34). While we were surprised by the substantial proportion of patients still FDA positive at 5M, at this point we have no evidence to suggest to extend the treatment duration for L6 patients. In Benin L5/6 were underrepresented among retreatment cases, with a prevalence of 12% among retreatment cases compared to 33.7% in newly infected patients[11,27].

When comparing all lineages (Fig 2C and 2D), L2 appears to convert the slowest, yet the sample size is insufficient to identify a significant difference. Similar studies in areas where L2 is highly endemic would be interesting to test whether L2 indeed converts significantly slower.

Differential susceptibility to TB drugs in vivo is not supported by drug susceptibility patterns in vitro. Unlike its close relative *M*. *bovis*, which is intrinsically PZA resistant due to a lineage wide *pncA* mutation, L6 is susceptible to PZA. However, L6 grows slower in vitro and is less dependent on aerobic metabolism[28], both of which may increase its tolerance to certain TB drugs like isoniazid that mainly target rapidly dividing bacteria [3,29]. In support of the slower response to TB treatment of L6, a study in The Gambia showed slower immunological recovery of patients treated for L6 relative to L4 [16].

Our data showed that a higher BMI at day 0, and HIV negative status were statistically significantly associated with shorter duration to smear conversion (Tables 2 and 3). This is different from Huenis in South Africa who found that HIV negative patients, mainly males, were less likely to convert at the end of 2M intensive phase [30]. Because of the limited resource setting with a high incidence of TB and patients presenting with advanced disease, the national TB program could consider increasing nutritional supplements in the fight against TB in Mali.

The strengths of our study include the prospective population based design in a setting where all main six MTBc lineages outside of Ethiopia are present, with prevalence estimates of L6 in line with our 2012 finding.

Our study has some limitations. We identified a relatively large proportion of unclassifiable spoligopatterns, mostly with many spacers present and without lineage specific signature pattern. We suspect that a proportion of these are reflective of mixed infection, as 24-locus MIRU-VNTR on a subset (S1 File) revealed double peaks in more than one locus in five of 19 lysates tested with a minimum of 10 loci successfully amplified, yet in-depth analysis of this group is beyond the scope of the present analysis. Such mixed infection is unlikely due to laboratory contamination, as culture was done according to accredited protocols designed to minimize cross contamination, and negative controls included in molecular analyses were consistently negative. Another limitation is that we only excluded based on *rpoB* mutations, and did not test for resistance to isoniazid, which may have differed by lineage and may have affected smear conversion rates. Our high rates of FDA positivity, despite double reading and quality control, may reflect an ‘overcalling’ of FDA positives with its fainter fluorescence than auramine. While we have no indication to believe that artefacts were identified as FDA positive bacilli, we expect that the comparison of conversion between lineages is valid regardless, as the microscopy readings were blinded to the spoligotype results. We did not follow patients for relapse, to test whether patients with L6 are indeed less likely to experience relapse, as would be suggested by its lower rate of primary progression after initial infection.

## Conclusions

This prospective study provides the first ‘unbiased’ population structure of MTBc in Bamako, where L4 predominates, similar to other West African countries. All six MTBc lineages identified outside of Ethiopia were also found in Bamako, with a substantial proportion of ancestral lineages (L1, L5 and L6, plus *M*. *bovis*, combined amount to 26.7%). We found that patients infected with L4 convert earlier than those infected with L6. In addition we found that the HIV negative status, higher BMI at day 0, and patients with smear grade at baseline ≤ 1+ were associated with earlier smear conversion.

## Supporting information

S1 File 24 locus-MIRU VNTR result of some isolates tested. Data are shown together with the positive and negative control results.
(XLSX)

S1 Checklist STROBE checklist. The Checklist used for describing the whole study.
(PDF)
